# Effect of Community-Based Functional Aerobic Training on Motor Performance and Quality of Life of Children with Spastic Cerebral Palsy

**DOI:** 10.4314/ejhs.v31i2.21

**Published:** 2021-03

**Authors:** Osei Evans Owusu Ansa, Kwadwo Wisdom Mprah, Monday Omoniyi Moses, Isaac Owusu, Enoch Acheampong

**Affiliations:** 1 Department of Health Education, Promotion and Disability Studies, School of Public Health, College of Health Sciences, Kwame Nkrumah University of Science and Technology, Kumasi, Ghana; 2 Department of Physiotherapy and Sports Science, Faculty of Allied Health Sciences, College of Health Sciences, Kwame Nkrumah University of Science and Technology, Kumasi, Ghana

**Keywords:** Exercise programme, Spastic CP, Walk distance, Gross motor function, Quality of life

## Abstract

**Background:**

Efficacies of community-based exercise programmes have been well reported, but there is scarce information on the expediency of community-based rehabilitation in a society where many children with disabilities live in poorly resourced settings with extremely limited rehabilitative services. This study investigated the effects of community-based functional aerobic exercise (CBFAE) on gross motor function, walking distance, and quality of life of children with cerebral palsy (CP).

**Methods:**

Quasi-experimental design was used. Children with gross motor function classification system (GMFCS) levels I – II participated in eight weeks CBFAE training four times/week, 50 minutes/day at 40–80% maximum heart rate. Gross motor function (GMF), walking distance and quality of life were assessed pre and post CBFAE training.

**Results:**

Significant improvement was observed in GMF (Dstanding) (8.2%, P=.000), GMF (E-walking + running+ jumping (5.12%, P=.004), walking distance (6.09%, P=.009). Higher significant positive effects of CBFAE were observed in Social wellbeing and acceptance (107.10%, P=.000), and participation and physical health (105.04%, P=.005) by children parent proxy. Self-reported results showed that for CBFAE, significant positive improvements were higher in pain and impact of disability (67.93%, P=.049) and participation and physical health (60.00%, P=.042).

**Conclusion:**

CBFAE training contributes majorly to improved standing, walking, jumping and running and self-esteem, quality of life of children with spastic CP. Clinicians and exercise therapists should essentially incorporate CBFAE training and activities into the management of children with CP for improved mobility and functional performances.

## Introduction

Cerebral palsy (CP) is a permanent but non-progressive damage to developing brain leading to permanent voluntary motor control and movement disorders in one or more limbs and sometimes in the trunk (1,2,3). CP commonly contributes to motor disability and serves as principal cause of physical disabilities in children (4). CP affects a person's boney structure (paralyzed limbs), motor functions/activities (grasping) and participation (playing tennis) which in turn may lead to activity limitations and participation restrictions (5).

Incidence of CP ranges from 1.5 to more than 4 per 1,000 live births worldwide. Although exact figures are unknown, it was speculated that 1 out of 300 children in Ghana are living with CP (6). Spastic is the most occurring subtype of CP present in about 80% of all cases (7). Spasticity negatively impacts activities of daily living such as dressing, feeding, grasping writing, walking, and bathing (8). Chronic spasticity leads to musculoskeletal disorders such as muscle pain, bone deformation, contractures, and dislocations which further limit functional independence, participation in meaningful activities and overall gross motor functioning (9). Motor functionalities in children with CP are classified using the gross motor function classification system (GMFCS). The GMFCS describes five levels of activity/functions: those in levels I and II walk without aids, those in level III walk with aids, and children in levels IV and V are nonambulatory. Gross motor development is a function physical activity in children with CP (10). Gross motor functions involve the use of large muscle groups in activities like rolling, sitting up, crawling, walking, jumping, running, hopping, leaping, and skipping skills are the ability to perform controlled movements (11).

Most children with spastic CP rarely show interest in active physical activities due largely to decreased motor control (12). Effects of exercise on spasticity grade have been studied (13) as well as effects of exercise on muscular strength in children with CP (9,11).

Lower exercise capacity and higher oxygen cost for activity are directly associated with children with CP (14,15). Aerobic exercise has been shown to reduce skin fold body fat, to improve peak oxygen consumption, ventilation and heart rate (16), to improve gross motor function and walking distance (17), aerobic capacity, quality of life and body composition (14) in children with CP.

Children with CP are unable to perform daily tasks, engage in leisure activities, enrol in school and participate in community activities. A further rather debilitating condition is that all or most children with CP are potentially sedentary and so prone to chronic cardiovascular diseases (CVDs), a pandemic health issue (18).

Despite numerous studies on the efficacy of community-based exercise programmes (19, 20), studies that used aerobic exercise therapeutically on children with CP have been conducted mainly in clinical and laboratory settings. Although there has been consensus among researchers and clinicians on the positive exercise effects have on children with CP (21), there are scarce studies in Ghana documenting the effects of community-based functional aerobic exercise (CBFAE) on gross motor function, walking distance and quality of life (QoL) of children with CP. The scarce information, to the best knowledge of the authors, suggests a possible disregard for the expediency of community-based rehabilitation in a society where the majority (80.0%) of children with disabilities live in resource poor settings. Additionally, society with extremely limited rehabilitative services will require active community-based fitness programmes for the vulnerable such as children and adults with CP to have desirable quality of life. Therefore, the present study investigated the effects of community-based functional aerobic exercise (CBFAE) on gross motor function, walking distance and quality of life of children with CP.

## Materials and Methods

**Study design**: The study followed a quasi-experimental design with pre-and post-training assessments. Measurements were conducted once before and after exercise intervention was introduced to the participants. Unlike the randomized controlled trials, quasi-experimental design deviates from popular random assignments.

**Participants**: The study population were 32 children with disabilities (intellectual disabilities-8, cerebral palsy-20, and down syndrome-4) between 11 and 17 years enrolled in the CoDREC at Manso-Atwere in the Amansie West District of the Ashanti Region of Ghana. Intellectual disabilities and down syndrome were referenced in this study to indicate the total population of children with chronic brain injury motor disorders in the study site. The non-probability purposive sampling method was used to recruit all the children with CP (20) which represents 70.0% of the total sample population for the study. Participants were included based on highest occurrence (70.0%) as affirmed in literature (7,22), medically diagnosed of spastic CP, had no other medical complications, walked with or without aids, could follow simple verbal instructions, and functional level was between GMFCS I-II. Participants were excluded when living with uncontrolled seizures, had cardiac diseases, undergone serial casting of the lower limb within the past three months, cannot follow simple verbal instruction and had GMFCS level was between III-V. Fourteen were eventually enrolled, but due to subject attrition, data from ten (10) participants who completed the CBFAE training were analysed for the study.

**Measurement**: Demographic information of gender, age (year), weight (kg), height (cm) and CP types were collected. Although not a focus of the study, values of weight (kg) divided by the square of height (m) were used to calculate body mass index (BMI) as a determinant of participants' obesity level.

**Gross motor function**: Changes in gross motor function (GMF) of the participants were assessed using the gross motor function measure (GMFM) - a clinical assessment tool designed to evaluate changes in gross motor function in children with cerebral palsy (CP). GMFM has two versions (GMFM-88 and GMFM-66) based on the number of items on each. Given that GMFM-88 has ordinal unidimensional scale and GMFM-66 has interval unidimensional scale of gross motor function, studies consistently established that the latter enhances scoring, interpretation, and observational clinical and research outcomes better than the former (23–24). Hence, this study used GMFM-66 developed for children with CP only and has tasks divided into five dimensions; (A) lying and rolling, (B) sitting (C) crawling and kneeling, (D) standing, (E) walking, running, and jumping. Each of the dimensions has item scored on a four-point scale (Table 1). The consistency, stability, and dependability of the GMFM-66 have been established over the years (24, 25) where interclass correlation coefficients [ICC] = 0.952- 1.00, standard error of measurement [SEM] and smallest real difference [SRD] of 1.60 and 3.14 respectively with interrater reliability of 0.93 and intra-rater reliability of 0.99–1.00.

**Table 1:** Scoring key for GMFM-66

Point	Interpretation
0	Does not initiate task
1	Initiates task (<10% of task)
2	Partially completes task (10 to < 100% of task
3	Completes task (100% of task)

[Waters et al., 26]

At the preliminary stage of identifying the difficulty level of the participants, all participants were able to execute three dimensions of GMFM-66 [(A) lying and rolling, (B) sitting (C) crawling and kneeling] spontaneously but could hardly perform (D) standing, (E) walking, running, and jumping. Furthermore, since GMFM-66 allows authors to measure particular items only (item maps), given that at least 13 items were included, 27 items in D and E dimensions of GMFM-66 were assessed and computed for this study.

**Walking distance**: The 30 Seconds Walk Test (30SWT) was used to assess walking distance of the participants. The 30SWT is a single-dimension test that does not only assess walking ability but also assesses coordination and exercise capacity associated with walking ability over a given distance with a given time. The reported standard protocol for using 30SWT (28) was adhered to as test was conducted on an oval even surface tartan track. Participants were asked to walk at own speed for 30 seconds. The total distance walked was measured to the nearest metre by means of attached meters marked on the track. The participants received verbal motivation and encouragement every 10 seconds. Where a participant experienced any pain and/or leg fatigue, fear of fall or, imbalance, he/she was permitted to slow down, stop or rest as necessary, but resumed walking as soon as possible. Three measurements were recorded and the mean of the three was taken.

**Quality of life**: Quality of life (QoL) instruments are accepted as standards for determining change in QoL in individuals. The instruments are of two versions: generic and condition specific (29). The generic instruments focused on wider perspectives of QoL and are used in general populations such as KIDSCREEN-10 (30) and child health questionnaire (31). The condition-specific instruments are appropriate to one group or specific disease condition and they are effective in detecting changes in a condition such as cerebral palsy quality of life questionnaires (CPQoL) (26, 32). CPQoL appealed to the interest of authors because a study showed that it is divided into self -report (57 items) and parent-proxy (66 items) versions with subscale components of Social well-being and acceptance (11 items), functioning (12 items), participation and physical health (11 items), emotional well-being (6 items), access to services (5 items), pain and impact of disability (8 items) and family health (4 items) (26, 32). Access to services and family health were, however, excluded for the self-reported as advised (26,32). The reliability and validity (Cronbach's alpha) of the CPQoL questionnaires have been ascertained (26,32). Reported internal consistency of CPQoL ranges between 0.63–0.93 for parent-proxy and 0.61 to 0.92 for child self-reports (26,32). Participants were able to understand and respond to the instrument in English language as official language in Ghana, hence CPQoL was not validated. The responses were scored on a 9-point scale, where 1 and 2 = very unhappy, 3 and 4 = unhappy, 5 and 6 = both neither happy nor unhappy, 7 and 8 = happy, and 9 = very happy. The scores were transformed to a scale with a possible range of 0 – 100 and were recoded.

**Intervention**: A functional aerobic exercise which could easily be implemented in the natural environment (school and community) was developed. All the participants performed the same exercises like running and changing direction of the body, kicking, step-ups, walking, bending and crouching. These tasks, aimed to improve daily functioning and mobility, were repeated throughout the programme. Exercise sessions were conducted in group and individualised approaches (all participants present) and supervised by professional exercise physiologists.

**Figure 2:**
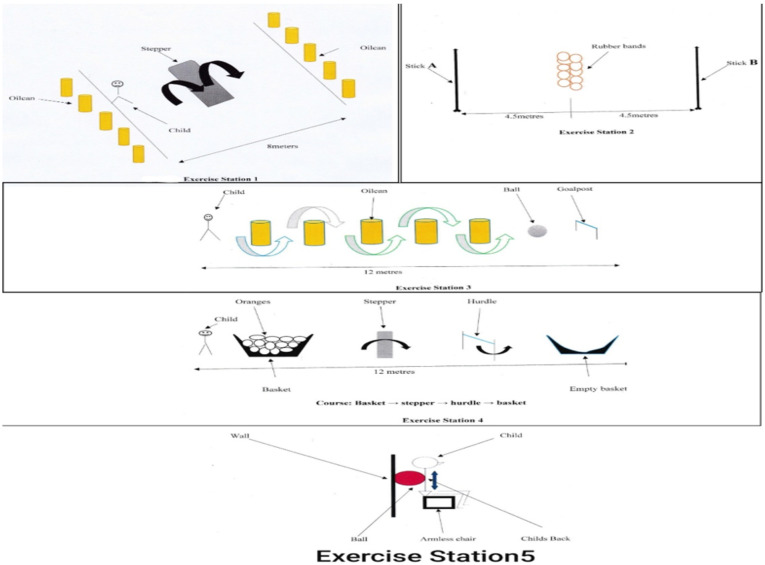
Exercise stations 1–5

CBFAE was conducted four times a week for eight weeks, at 40% – 70% of maximal heart rate (MHR) using circuit training principle. The circuit training programme employed had five exercise stations (Figure 2) with one aimed at increasing strength of larger muscle groups of both lower limbs and the other four aimed at increasing aerobic endurance.

Exercise Station 1 consisted of ten oilcans and one stepper (block). Five oilcans were placed at both ends of a stepper (block) of 4 meters apart. The activities conducted were running, hopping and pushing, aimed to increase aerobic endurance of the children. The participants at one end ran forward, stepped over the stepper, knocked an oilcan, ran back, stepped over the stepper again and knocked over another oilcan till all the oilcans were knocked down.

Exercise Station 2 comprised of two wooden sticks (A and B) of 2 meters long and 15 rubber bands. The wooden sticks were placed vertically equidistance opposite each other at 9 metres. The rubber-bands were placed on level ground (floor) at the center (4.5 meters) of the sticks. Participants required to run, bend and stand, aimed at increasing aerobic endurance. The participants ran from stick (A), picked up only one rubber-band, moved toward and put it around the stick (B), then ran from stick B, picked another one rubber-band, moved toward and placed it around stick A. This process continued until all the rubber-bands were placed on the sticks.

Exercise Station 3 consisted of five oilcans, a ball and a goal post. The five oilcans were arranged on a straight line, one meter apart within the distance of 12 metres. A ball was placed one meter after the last oilcan and before the goal post. The activities at this station involved running, twisting and kicking, aimed at increasing aerobic endurance. Starting one meter away from the first oilcan, the participants ran between the oilcans and kicked a ball into the goal post with the aim of scoring. One person acted like goalkeeper to guard the goal post and prevented the ball from entering the goal. The goalkeeper then put the ball back in place after each kick. This process was repeated for five times.

Station 4 consisted of 10 oranges, 1 stepper, 1 hurdle stand and 2 baskets. One basket containing the 10 oranges and the other basket (empty) were placed 12 meters apart from each other. The stepper and the hurdle stand were placed 3meter apart and between the baskets. Activities involved were bending, running, hopping, and crouching, aimed at increasing aerobic endurance. The participant started behind the basket with oranges, took one orange from the basket, ran and stepped over the stepper (block), ran and bend under the hurdle stand, and placed the orange in the empty basket after the hurdle stand. The process is repeated till all the oranges were transferred.

Station 5 consisted of an armless chair, a wall, and a big ball. The armless chair was placed near the wall. The participant sat on the armless chair with back against the FIFA standard sized ball glued to the wall. Activities involved building muscles at the thigh, leg, gluteus and arms to improve muscular strength. Participants moved from sitting (knees 90 degrees flexion) to standing position (knees 180 degrees extension) and back to sitting position resting on the ball. The participants repeated this exercise for 10 times. In total, each child performed three sets of five repetitions with the ball guiding each movement (35).

Participants warmed-up and cooled-down for ten minutes respectively prior to and at the completion of each session. The exercise involved stationary marching, stretching of the thigh and calf muscles of both lower limbs. Participants trained at each station for 5 – 6 minutes totaling 30 minutes, 2 – 3 minutes of rest amounting to 10 minutes and then moved to the next station following completion of each exercise. During the first day of training, participants were made to familiarize themselves with the equipment, and appropriate intensity of exercise was determined. The intensity of the aerobic exercise was determined using the Karnoven's formula that shows age predicted heart rate (220 minus age in years). Karnoven's formula has been reported to significantly predict exercise intensity (36,37). New intensity for aerobic training was determined on the basis of resting heart rate every week.

The community-based functional aerobic exercise (CBFAE) training was made captivating and interesting for the participants. CBFAE involved actions of daily activities and local equipment to increase motivation and willingness to continue even when the training period has ended. To make the training interesting and yet provide the needed aerobic benefit and adherence, it was varied by integrating sporting activity including goal ball to increase the participants' self- esteem as many children without disability eager to participate.

**Statistical analysis**: The data that were entered into excel spreadsheet contained the pre- and post-test scores and the paired difference. Paired sample t-test was used to compare differences in pre- and post-test scores. Statistical significance level was set at P < 0.05 while calculated test statistics is greater than the critical value at 95% confidence level.

**Ethical consideration**: Ethical Committee on Human Research Publication and Ethics (CHRPE), KNUST, reviewed and approved the study protocol and implementation technique (CHRPE/AP/489/17). The Community-Based Rehabilitation (CBR) programmes manager granted the permission to conduct the research at Community Day Rehabilitation and Education Centre (CoDREC) after the objectives and rationale of the study had been explained to her. Confidentiality of participants' information was assured throughout the study. A consent form was completed by participants/parents and all procedures and tests were also clearly explained to participants and parents.

## Results

**Demographic information**: The mean age of the participants was 14.4 years (range 12–16years), mean height was 146.5cm (range 139–157cm), and mean weight was 48.9 kg (range 40–58 kg). Four (4) of the participants were diplegic, one (1) was hemiplegia and five (5) were quadriplegia spastic. The participants had normal weight seen from body mass index value (22.94kg/m^2^) (Table 2).

**Table 2:** Demographic characteristics of the study participants

Variable		Character	Frequency(f)	Percentages (%)
Gender	Male		7	70.0
	Female		3	30.0
	< 13		2	20.0
Age (year)	13 -14		2	20.0
	15 -16		6	60.0
	Mean (SD); Min-Max		14.4(1.53); 12–16	
	≤ 40		1	10.0
	41 – 50		5	50.0
Weight(kg)	51 – 60		4	40.0
	Mean (SD); Min-Max		48.9(6.24); 40–58	
	≤ 140		2	20.0
	141 – 150		6	60.0
Height(cm)	>150		2	20.0
	Mean (SD); Min-Max		146.5(61.32); 139–157cm	
Type of CP	Diplegia		4	40.0
	Hemiplegia		1	10.0
	Quadriplegia		5	50.0

**Motor performance**: Community-based functional aerobic exercise (CBFAE) significantly improved in GMF (D- standing) by 8.2% (*P*=.000), GMF (E-walking, running, jumping) by 5.12% (*P*=.004) and walking distance by 6.09% (*P*=.009) (Table 3).

**Table 3:** Pre-post-test comparison of motor performance and quality of life

Variables	Mean ± SD		Mean Diff (% of change)	*t*	p value

	Pretest	Post test			
GMF-(D)	63.23±2.70	68.42±3.92	5.19±3.45(8.2)	4.753	0.000*
GMF-(E)	59.57±1.98	62.62±3.60	3.05±2.54(5.1)	3.812	0.004*
30 Second Walking	197.10±12.91	209.10±18.44	12.00±8.73(6.9)	8.730	0.009*
Parent Proxy Report					
Participation and physical health	25.00±10.20	51.25±11.86	26.25±5.31(105.04)	3.279	0.005*
Emotional well-being	34.00±09.02	55.32±18.42	21.25±09.40(62.50)	4.352	0.015*
Pain and impact of disability	22.07±2.37	42.41±4.65	20.34±2.28(92.16)	3.624	0.024*
Functioning	17.50±10.54	30.00±16.46	12.50±9.72(71.43)	2.875	0.009*
Access to services	46.25±11.86	65.00±15.37	18.75±4.73(40.54)	4.020	0.002*
Social wellbeing and acceptance	23.38 ±4.50	48.42±3.29	25.04±3.45(107.10)	4.533	0.000*
Family health	27.33±15.19	41.13±14.46	13.80 ±1.53(50.49)	3.221	0.044*
Teen Self-Reported Social wellbeing and acceptance	30.00±12.70	46.25±14.46	16.25±8.89(54.17)	1.770	0.060
Participation and physical health	21.25±14.16	34.00±11.30	12.75±2.86(60.00)	3.012	0.042*
Pain and impact of disability	26.38±18.52	44.30±12.12	17.92±6.12(67.93)	3.101	0.049*
Emotional well-being	33.75±16.71	48.75±22.40	15.00±1.08(44.44)	2.250	0.025*
Functioning	43.75±14.73	60.00±16.45	16.25±1.29(37.14)	2.410	0.019*

* Significant at 0.05

**Quality of life**: Highest significant positive effects of CBFAE was observed in social wellbeing and acceptance (107.10%, *P*=.000) followed by participation and physical health (105.04%, *P*=.005) while access to services received the least significant improvement (40.54%, *P*=.002) as reported by children's parent through proxy. Self-reported results showed that CBFAE significant positive improvement was highest in pain and impact of disability (67.93%, *P*=.049) followed by participation and physical health (60.00%, *P*=.042) whereas functioning (37.25%, *P*=.019) was the least. It was further observed that there was no significant effect of CBFAE on social wellbeing and acceptance (54.17%, *P*=.060) based on teen self-report (Table 3).

## Discussion

This study investigated the effects of community-based functional aerobic exercise (CBFAE) on gross motor function, walking distance, and quality of life (QoL) of children with cerebral palsy (CP). The findings indicated that 8-weeks CBFAE improved gross motor functions, walking distance and QoL of CP children. These findings substantiate the submission on exercise regimen being the most effective interventions for CP management (1,2,3). Specifically, with effects of 8-weeks CBFAE on gross motor functions of standing, walking, running and jumping, there was significant improvement. Similar improvement was reported in the standing, walking, running and jumping gross motor function of children with mild cerebral palsy using group-task-oriented training (38) although greater postural stability was not shown after eight weeks. This suggests positive effects of the community-based functional aerobics training on the standing, walking, running and jumping gross motor function on CP children. A quasi - experimental study conducted by Rahman et. al., (17) showed that a six-week circuit training improved static and dynamic gross motor function of standing, jumping, running and walking. Akbas and Gunel's (39) study further established significant improvement in motion functions in all dimensions of the experimental group. Persistent practicing of functional activities is seen to improve primitive motor reflexes which enhanced movement in conscious and unconscious pathways and decrease fall tendencies in non-ambulant children with cerebral palsy (40). Equally observed with the study of Bania et al., (10) was improvement in gross motor functions of children with CP following a six-weeks exercise intervention.

Another finding of the present study was the significant improvement in walking distance of children with CP following eight-weeks CBFAE. This finding on the walking distance is in accordance with the submission of Teixeira et al. (41) who evaluated the effects of cardiovascular training of individual with CP where significant improvement was found in walking distance and speed. Similarly, significant improvement was observed in walking distance at the end of an eight-week study to investigate the effects of aerobic exercise on children with CP (1,2,5,13,35,40).

The significant improvement in walking distance observed in the present study might be associated with training duration, intensity and its community-based nature. In sedentary and/or inactive individuals, training result in significant improvement in exercise capacity in that, it elicits physical strain on the individual as a result of the increase in energy expenditure. The system then develops structural and functional adaptations to accommodate the change in expenditure which enables it to deliver more oxygen (O_2_) and nutrients and to get rid of carbon dioxide (CO_2_) and metabolic waste products thereby improving the efficiency of the cardiorespiratory system (14,15). Moreover, the functional nature of the training programme ensured that the force, torque and momentum generated by the muscles (calf, gluteus, knee extensor and flexor) are directly related to walking hence the positive results. The functional training has positive effects on motor aspects such as walking and endurance hence the improving mobility and functions performance.

The final findings of this study centered on the significant improvement in all the domains of the parent-proxy version and less one in the teen-self reported version of the QoL domains after eight weeks CBFAE. Following an eight-week case study to evaluate the effect of cardiovascular training on adult with athetoid CP, Teixeira et al., (41) observed a significant improvement in the QoL. The findings of this study substantiate positive outcomes of several studies where effects of aerobics training on the QoL of children with CP were investigated (2,5,14,35,41). Personal interest and opportunity to participate in activities is an important contributor to QoL. Improvement in the QoL as reported by participants might be due to the nature of activity, setting and personal interest.

Eight weeks community-based functional aerobic exercise (CBFAE) effectively improves gross motor function, standing, walking, jumping, running and walking distance of children with spastic cerebral palsy (CP). CBFAE positively enhances QoL – parent-proxy and teen self-reported versions. Clinicians and exercise therapists should essentially incorporate CBFAE training and activities into the management of children with CP in general and spastic CP specifically for improved mobility and functional performances.

This study obtained data from children between 11–17 years whose ideas might sometimes be underestimated or seen to be underage. Although parents' information was also obtained, children information sometimes could serve as limitation in knowledge provision for dissemination. Aside the aforementioned limitation, the sample size used within specific stratum of Ghana needs to be considered when using the outcome of the study. It is also germane to indicate lack of control group in the study. To significantly enhance and influence positive national community-based functional aerobic exercise policy decision for children with CP, funded nationwide longitudinal study using the protocol in this study is recommended.

## Figures and Tables

**Figure 1:**
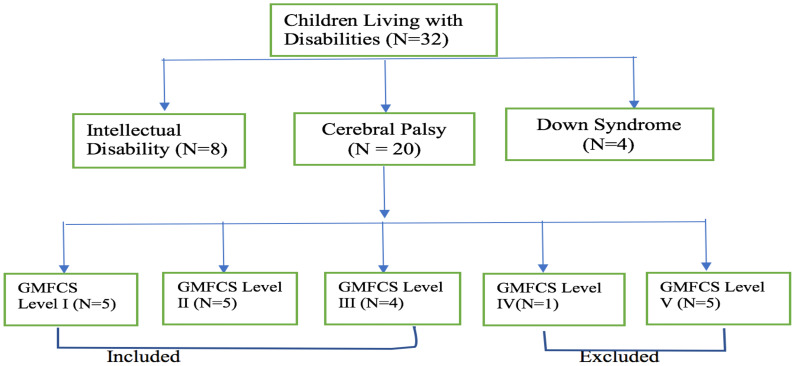
Flowchart diagram
